# Is synovial hypertrophy without Doppler activity sensitive to change? Post-hoc analysis from a rheumatoid arthritis ultrasound study

**DOI:** 10.1186/s13075-018-1709-6

**Published:** 2018-10-03

**Authors:** Lene Terslev, Mikkel Østergaard, Joe Sexton, Hilde Berner Hammer

**Affiliations:** 1 Copenhagen Center for Arthritis Research and Center for Rheumatology and Spine Diseases, Centre of Head and Orthopaedics, Rigshospitalet, Copenhagen, Denmark; 2 Department of Clinical Medicine, University of Copenhagen, Copenhagen, Denmark; 3 Department of Rheumatology, Diakonhjemmet Hospital, Oslo, Norway

**Keywords:** Ultrasound, Synovitis, Rheumatoid arthritis, Doppler, Sensitivity-to-change

## Abstract

**Background:**

To explore to what extent synovial hypertrophy in joints *without* Doppler activity is a sign of active disease, we investigated the sensitivity to change of synovial hypertrophy *without* Doppler activity during biological disease-modifying antirheumatic drug (bDMARD) treatment in rheumatoid arthritis (RA) patients.

**Method:**

RA patients initiating or switching bDMARD treatment had ultrasound (US) performed on 36 joints at baseline, and at 3 and 6 months. Synovial hypertrophy by grayscale US and Doppler activity were graded separately from 0 to 3 at the joint level for all time points. Changes in synovial hypertrophy in joints without Doppler activity during treatment were assessed and compared with changes in synovial hypertrophy in joints with Doppler activity.

**Results:**

We included 151 patients (82.8% women, 80.1% seropositive for anticyclic citrullinated peptide) with a mean ± standard deviation age of 51.4 ± 13.2 years, a disease duration of 9.9 ± 7.9 years, and baseline Disease Activity Score 28-joint count C-reactive peptide (DAS28-CRP) of 4.14 ± 1.32. At baseline, 44.8% of all joints examined (*n* = 5225) had synovial hypertrophy ≥ 1 and 50.7% of these had synovial hypertrophy *without* Doppler activity. The improvement in synovial hypertrophy was similar in joints with and without Doppler activity but, when adjusting for the baseline score of synovial hypertrophy, joints with synovial hypertrophy *without* Doppler had a higher tendency towards a decrease than joints with synovial hypertrophy *with* Doppler activity independent of grade (3 months: *p* < 0.0001; 6 months: *p* = 0.0003).

**Conclusion:**

Joints *with* synovial hypertrophy *without* Doppler activity improve during treatment, independent of the grade. Thus, SH *without* Doppler activity is not a sign of inactive disease. These findings indicate that joints *with* synovial hypertrophy *without* Doppler activity should also be taken in to account when assessing disease activity by US.

## Background

The treatment strategy and treatment options for rheumatoid arthritis (RA) have improved considerably over the past years. The strategy is to “treat-to-target” (with a target of remission or low disease activity (LDA)) and includes early and aggressive treatment with mono- or combination therapy using disease-modifying antirheumatic drugs (DMARDs). Furthermore, rapid treatment escalation is recommended when there is insufficient clinical response, including the addition of biological DMARDs (bDMARDs). The aim of this strategy is to gain rapid disease control and avoid inflammation, thereby preventing pain and joint destruction and improving physical function [1, 2].

In addition to conventional clinical monitoring of treatment in RA patients, ultrasound (US) has been increasingly applied over the last decade to assess disease activity and the response to therapy. As US has been proven to be more sensitive than clinical joint evaluation [3, 4], the addition of US to clinical joint evaluation has allowed for a more elaborate joint involvement, helping in correctly classifying and diagnosing RA patients [5]. When assessing synovitis by US, two components are evaluated: synovial hypertrophy by grayscale (GS) US and hyperemia by Doppler US. Scoring systems have been developed for the two components, with each scored individually [6–10]. Most emphasis in the clinical studies/trials has been on changes in Doppler activity as this is considered to reflect the inflammatory activity, i.e., disease activity [10–12], and hence the primary aim has been the elimination of signs of Doppler activity [13–17].

Joints with synovial hypertrophy *without* Doppler activity are generally believed to be inactive changes [12] and without the ability to change when optimizing treatment [18], thereby being of minimal clinical importance. However, very little is known about how GS synovial hypertrophy *without* Doppler activity behaves in joints during treatment, i.e., if these joints have the potential to improve. The fact that GS synovial hypertrophy per se (though weaker than Doppler activity) is a predictor of erosive progression in patients in remission [19, 20] suggests that GS synovial hypertrophy is important and possibly should be part of imaging remission, and that elimination of Doppler per se is potentially not the only treatment goal in RA patients when considering treatment escalation.

It is therefore of great importance to explore the reversibility of synovial hypertrophy in joints *without* Doppler activity as this—if sensitive to change—may impact decisions to optimize or change treatment in both clinically active RA patients and in patients in remission/LDA.

The aim of the present paper is to explore if synovial hypertrophy in joints *without* Doppler activity is sensitive to change, as compared with synovial hypertrophy in joints *with* Doppler activity, when initiating or switching treatment with bDMARDs in patients with RA. Furthermore, the relationship between the presence of synovial hypertrophy *with and without* Doppler activity and clinical evaluation of disease activity is investigated.

## Methods

The study cohort comprised of patients with RA fulfilling the American College of Rheumatology (ACR) 1987 criteria [21] initiating or switching bDMARD treatment in the period from January 2010 to June 2013 (Anzctr.org.au identifier, ACTRN12610000284066). Clinical evaluation (number of tender and swollen joints (= 28), patient and assessor global visual analogue scale (VAS), C-reactive protein (CRP), and US examinations of 36 joints) were performed at baseline, and at 3 and 6 months. Two trained study nurses with longstanding experience in evaluating tender and swollen joints performed the clinical evaluations blinded to the US findings. Disease Activity Score 28-joint count (DAS28)-CRP was calculated for each visit.

US examinations (blinded for the results of the clinical assessments) were performed by the same experienced sonographer (HBH) throughout the study using a Siemens Antares, Excellence version (Siemens Medical Solutions, CA, USA) equipped with a 5–13 MHz linear probe. Power Doppler settings were optimized for inflammatory flow with pulse repetition frequency 391 Hz, Doppler frequency 7.3 MHz, and gain just below the level of noise according to guidelines [22]. The same ultrasound machine with the same settings was used throughout the study and no software upgrade was done.

At each visit, US examination for signs of synovitis was performed for the following 18 joints bilaterally: metacarpophalangeal joints 1–5, proximal interphalangeal joints 2–3, wrist (scoring radiocarpal, intercarpal, and radioulnar joints separately), elbow, knee, talocrural, and metatarsophalangeal joints 1–5. Each joint at each visit was scored semiquantitatively from 0–3 for GS and power Doppler (PD) according to the US atlas of Hammer et al. [8]. Joints with recent surgery, prosthesis, or overlying skin infections were not examined at time of inclusion or during follow-up. The performing sonographer has previously demonstrated a high intra-observer reliability (weighted kappa 0.83 [8]) for the included joints using this atlas [8] for scoring.

The study was approved by the Regional Committee for Medical and Health Research Ethics, South-East Norway, and all patients gave written consent according to the Declaration of Helsinki.

A post-hoc analysis was carried out on those patients from the original cohort [23] fulfilling all follow-up visits. Only joints with follow-up at all time points were included in the analysis.

The primary endpoint of the study was to assess the relative frequency of a synovial hypertrophy score change ≥ 1 in joints *without* Doppler activity versus joints *with* Doppler activity in all joints having a baseline synovial hypertrophy of grade 1 or above. The changes were assessed from baseline to 3-month follow-up and baseline to 6-month follow-up. The secondary endpoint was the correlation between the change in mean synovial hypertrophy score in joints *without* Doppler activity and the change in mean synovial hypertrophy score in joints *with* Doppler activity in joints with baseline synovial hypertrophy of grade 1 or above. This was assessed from baseline to 3-month follow-up and baseline to 6-month follow-up. The tertiary outcome was the association between the mean grade of synovial hypertrophy in joints and the presence/absence of clinical joint tenderness and presence/absence of clinical joint swelling in joints as follows: 1) at 3 months in joints with baseline Doppler activity; 2) at 6 months in joints *with* baseline Doppler activity; 3) at 3 months in joints *without* baseline Doppler activity; and 4) at 6 months in joints *without* baseline Doppler activity. Finally, we assessed the correlation between changes in mean synovial hypertrophy in Doppler-negative joints, Doppler-positive joints, and changes in DAS28-CRP from baseline to 3-month follow-up and baseline to 6-month follow-up.

### Statistics

R statistical software version 3.3.2 was used for analyses. To assess differences in the tendency of synovial hypertrophy to decrease between Doppler-positive versus Doppler-negative joints, the corresponding odds ratio between these two groups was estimated. This was performed using a logistic mixed-effects model, where the dependent variable was decrease in synovial hypertrophy from baseline ≥ 1 point, coded as a binary variable equal to 1 if a decrease was observed (for a given joint at a given time point) and 0 otherwise. The independent variable was Doppler status of the joint at baseline (0 if Doppler = 0, 1 if Doppler > 0), further adjusting for both baseline synovial hypertrophy level of each joint as well as the observation time (treated as a categorical variable). To account for dependencies between observations from the same patient, as well as from the same joint, random intercepts for both terms were included in the model. Only joints with synovial hypertrophy at baseline were included in the analysis, and the model was fit using data at both 3 and 6 months. The difference in mean grade synovial hypertrophy between tender and non-tender joints was evaluated using a bootstrap test. The same was performed for joint swelling. Correlation between change in mean synovial hypertrophy across joints and change in DAS28-CRP was calculated using Spearman’s correlation.

## Results

Of the original cohort of 212 patients [23], 151 completed both baseline and 3- and 6-month follow-up. Baseline demographics for the included patients are listed in Table 1.

**Table 1:** Demographic data for the 151 patients

Patients	82.8% women
Age (years)	51.4 ± 13.2
Anti-CCP	80.1% positive
Rheumatoid factor	75.2% positive
Disease duration (years)	9.9 ± 7.9
Number of swollen joints	6.49 ± 5.5
Number of tender joints	5.98 ± 6.4
bDMARDs initiated in the study cohort	Infliximab (*n* = 19), etanercept (*n* = 59), adalimumab (n = 8), certolizumab (*n* = 12), golimumab (*n* = 8), rituximab (*n* = 30), tocilizumab (n = 8), and abatacept (*n* = 5); 8% of the patients were biologic naive
Prednisolone treated	50.7%
Prednisolone dose	7.8 ± 4.68 mg
DAS28-CRP score	4.14 ± 1.32)
Total number of joints examined, *n*	5225
Joints with SH and no Doppler at baseline, *n* (%)	1191 (23%)
Joints with SH and Doppler at baseline, *n* (%)	1151 (22%)

Values are shown as mean ± standard deviation unless otherwise indicated

*bDMARD* biological disease-modifying antirheumatic drug, *CCP* cyclic citrullinated peptide, *DAS28-CRP* Disease Activity Score 28-joint count C-reactive peptide, *SH* synovial hypertrophy

In total, 5225 joints were evaluated at baseline and, of these, 1191 joints had synovial hypertrophy *without* Doppler activity but only 1172 had follow-up at both 3 and 6 months. At baseline, 1154 had synovial hypertrophy *with* Doppler activity, but only 1122 had follow-up at 3 and 6 months. The reasons that joints were unavailable for US examination at 3 and/or 6 months included recent surgery, including prosthesis surgery, joint trauma, or overlying skin infections.

Joints with synovial hypertrophy *without* Doppler activity had overall lower grades of synovial hypertrophy (1.3 ± 0.3 (mean ± standard deviation (SD)) than joints with synovial hypertrophy *with* Doppler activity (2.1 ± 0.5) at baseline. In general, joints with lower levels of synovial hypertrophy had lower tendency towards change (Table 2).

**Table 2:** Improvement in synovial hypertrophy (SH) in joints with and without Doppler activity

Time	Grades	Joints with SH without Doppler activity			Joints with SH with Doppler activity			Adjusted odds ratio	*p* value
		Number of joints		Frequency of improvement	Number of joints		Frequency of improvement		
		Baseline	With improvement		Baseline	With improvement			
3 months	All grades	1172	637	0.54	1122	594	0.53	0.35	< 0.0001
	Grade 1	863	441	0.51	169	58	0.35		
	Grade 2	269	164	0.61	494	271	0.55		
	Grade 3	40	32	0.80	459	265	0.58		
6 months	All grades	1172	657	0.56	1122	669	0.60	0.39	0.0003
	Grade 1	863	457	0.53	169	58	0.34		
	Grade 2	269	167	0.62	494	312	0.63		
	Grade 3	40	33	0.82	459	299	0.65		

The table shows the frequency of improvement in joints at 3 and 6 months for all grades overall and separately for each grade

The relative frequency of a SH score decrease ≥ 1 were analyzed using a generalized linear mixed model adjusting for baseline SH score using a separate coefficient per time point

### Improvement in synovial hypertrophy during treatment

The overall frequency of change in synovial hypertrophy in joints *without* Doppler activity and joints *with* Doppler activity was similar at both 3 months (0.54 and 0.53, respectively) and 6 months (0.56 and 0.60, respectively) (Table 2). However, when adjusting for the baseline grade of synovial hypertrophy, the change was significantly different between joints *with and without* Doppler activity. The adjusted odds ratios were 0.35 at 3 months and 0.39 at 6 months, showing that joints with synovial hypertrophy *with* Doppler activity were significantly less likely to change than joints with synovial hypertrophy *without* Doppler activity for all grades (3 months: *p* < 0.0001; 6 months *p* = 0.0003).

The mean ± SD synovial hypertrophy in joints *without* Doppler activity at both 3 and 6 months was 1.3 ± 0.3 with a change from baseline of −0.5 ± 0.4 and −0.6 ± 0.4, respectively. In joints with synovial hypertrophy *with* Doppler activity, the synovial hypertrophy score at both 3 and 6 months was 2.1 ± 0.5 with a change at 3 months of −0.7 ± 0.6 and at 6 months of −0.8 ± 0.6. The change in mean synovial hypertrophy score in joints *with and without* Doppler activity correlated significantly at both 3 and 6 months (*r* = 0.37, *p* < 0.0001, and *r* = 0.34, *p* < 0.0001, respectively).

Of the joints with synovial hypertrophy *without* Doppler activity included in the analysis, 43% had achieved a synovial hypertrophy score of 0 at 3 months and 45% at 6 months while, for the joints with synovial hypertrophy *with* Doppler activity, this was found in 20% and 24% at 3 and 6 months, respectively. The frequency of different synovial hypertrophy grades at different time points are shown in Table 3.

**Table 3:** The frequency of different synovial hypertrophy (SH) grades at different time points

Time	SH grade 0	SH grade 1	SH grade 2	SH grade 3
Baseline	53.9%	19.1%	14.2%	9.3%
3 months	61.1%	19%	11%	5%
6 months	61.6%	19.1%	10%	4.3%

The mean synovial hypertrophy grade in different joint groups were comparable (mean 1.23, range 1.15–1.53, at baseline in joints with synovial hypertrophy *without* Doppler activity). All joint groups showed an improvement in synovial hypertrophy at both 3 and 6 months compared with baseline (data not shown).

### Association between mean grade of synovial hypertrophy and clinical evaluation

#### Joint swelling

When evaluating the joints with synovial hypertrophy *without* Doppler activity there was a significantly higher grade of synovial hypertrophy in joints with clinical joint swelling compared with joints without clinical joint swelling at all time points (*p* < 0.0001). At baseline, the mean grade of synovial hypertrophy was 0.81 in clinical swollen joints as opposed to 0.22 in non-swollen joints; at 3 months it was 0.92 versus 0.19, and at 6 months 1.07 versus 0.20. Similar findings were seen in the joints with synovial hypertrophy *with* Doppler activity but with higher mean synovial hypertrophy score at all time points (2.56 in swollen versus 2.12 in non-swollen joints at baseline, 2.49 versus 1.95 at 3 months, and 2.53 versus 1.86 at 6 months).

#### Joint tenderness

When evaluating the joints with synovial hypertrophy *without* Doppler activity, there was a significant difference in mean synovial hypertrophy score in clinically tender joints compared with non-tender joints at all time points (*p* < 0.0001). At baseline the mean synovial hypertrophy score was 0.50 in clinically tender joints as opposed to 0.24 in non-tender joints; at 3 months the results were 0.44 versus 0.22, and at 6 months 0.48 versus 0.23, respectively. However, in joints with synovial hypertrophy *with* Doppler activity there was no statistically significant difference in mean synovial hypertrophy score between clinically tender and non-tender joints at any time point (2.45 in tender versus 2.45 in non-tender joints at baseline, 2.29 versus 2.31 at 3 months, and 2.38 versus 1.33 at 6 months).

#### Correlation between synovial hypertrophy and DAS28-CRP remission

There was a weak but statistically significant correlation between change in the mean synovial hypertrophy score across all joints and the change in DAS28-CRP for joints with synovial hypertrophy with as well as *without* Doppler activity. The correlation coefficient for joints with synovial hypertrophy *without* Doppler activity was 0.28 (*p* = 0.001) at 3 months and 0.17 (*p* = 0.03) at 6 months. For joints with synovial hypertrophy *with* Doppler activity, the correlation coefficient was 0.23 (*p* = 0.007) at 3 months and 0.34 (*p* = 0.001) at 6 months.

## Discussion

The present study reveals that joints with synovial hypertrophy *without* Doppler activity are sensitive to change during bDMARD treatment and, when taking into account the baseline score of the synovial hypertrophy, joints with synovial hypertrophy *without* Doppler activity have a higher tendency towards change than joints with synovial hypertrophy *with* Doppler activity, independent of the grade.

Even though the treatment effect is evaluated by both GS and Doppler, the main focus is often on the changes in Doppler activity since the Doppler activity is believed to reflect the inflammatory component of the disease [11, 12, 14, 16, 18]. Although the two components in synovitis (i.e., synovial hypertrophy and hyperemia) are likely to change in parallel when treatment is initiated [10, 24, 25], there has been a lack of evidence of the ability for joints with initial synovial hypertrophy without signs of hyperemia to change per se during treatment. In 2013, Witt et al. [18] showed that synovial hypertrophy grade 1 in cohorts of both early and late RA patients was less likely to respond to treatment and, furthermore, grade 1 was also found in healthy controls. These grade 1 findings were not associated with swelling or tenderness in either RA patients or healthy controls. Padovano et al. reported similar frequent findings of grade 1 synovial hypertrophy among healthy controls [26]. In the current study, we have demonstrated that, in RA patients, synovial hypertrophy both *with and without* Doppler activity has the ability to improve when initiating effective treatment, even grade 1 synovial hypertrophy. Similar results have been seen for tenosynovitis, where grade 1 tenosynovitis without Doppler improves during treatment [27]. The US changes were also compared with changes in DAS28-CRP (i.e., to clinical assessment of treatment response). A weak but significant correlation was found between changes in DAS28-CRP and changes in mean synovial hypertrophy score for both joints *with and without* Doppler activity, indicating that the improvements in the joints are in line with the overall disease improvement during treatment. Furthermore, in the current study, the mean grade of synovial hypertrophy was higher in joints *with* than *without* Doppler activity, both in tender and swollen joints, supporting that both measures reflect aspects of disease activity.

The present study shows that synovial hypertrophy is susceptible to change during treatment but does not indicate which of the two components is the most important. In a recent publication from the Outcome Measures in Rheumatology (OMERACT) US group [9, 28], a consensus-based scoring system for synovitis in RA patients has been developed taking both components of the synovitis complex into account in a combined score. The relevance of including the GS synovial hypertrophy in this scoring is further substantiated by the findings in the present study.

The impact of Doppler sensitivity on scoring has previously been investigated and it has been shown that suboptimal settings and suboptimal equipment may result in lack of Doppler activity in inflamed joints [29]. In the present study, high-end US equipment was used to make sure that the detected joints with synovial hypertrophy but no Doppler activity truly reflected a lack of hyperemia in the joints.

## Conclusion

Our study shows that joints with synovial hypertrophy *without* Doppler activity improve during bDMARD treatment, demonstrating that synovial hypertrophy *without *Doppler activity is a sign of active disease. These findings indicate that joints with synovial hypertrophy *without* Doppler activity should be taken into account when assessing disease activity by ultrasound.

## Abbreviations

ACR: American College of Rheumatology
bDMARD: Biological disease-modifying antirheumatic drug
CRP: C-reactive protein
DAS28: Disease Activity Score 28-joint count
DMARD: Disease-modifying antirheumatic drug
GS: Grayscale
LDA: Low disease activity
OMERACT: Outcome Measures in Rheumatology
RA: Rheumatoid arthritis
SD: Standard deviation
US: Ultrasound
VAS: Visual analogue scale
